# The insulin-degrading enzyme as a link between insulin and neuropeptides metabolism

**DOI:** 10.1080/14756366.2020.1850712

**Published:** 2021-01-05

**Authors:** Dorota Wacławczyk, Jerzy Silberring, Giuseppe Grasso

**Affiliations:** a Department of Biochemistry and Neurobiology, AGH University of Science and Technology, Krakow, Poland; b Department of Chemical Sciences, University of Catania, Catania, Italy

**Keywords:** Insulin-degrading enzyme, neuropeptides, diabetes, opiods

## Abstract

We have applied a recently developed HPLC-MS enzymatic assay to investigate the cryptic peptides generated by the action of the insulin-degrading enzyme (IDE) on some neuropeptides (NPs) involved in the development of tolerance and dependence to opioids. Particularly, the tested NPs are generated from the NPFF precursor (pro-NPFF (A)): NPFF (FLFQPQRF) and NPAF (AGEGLSSPFWSLAAPQRF). The results show that IDE is able to cleave NPFF and NPAF, generating specific cryptic peptides. As IDE is also responsible for the processing of many other peptides in the brain (amyloid beta protein among the others), we have also performed competitive degradation assays using mixtures of insulin and the above mentioned NPs. Data show that insulin is able to slow down the degradation of both NPs tested, whereas, surprisingly, NPAF is able to accelerate insulin degradation, hinting IDE as the possible link responsible of the mutual influence between insulin and NPs metabolism.

## 1. Introduction

Cryptic peptides are bioactive amino acid sequences hidden in a precursor protein, which are formed and released when a peptidase cleaves the parent protein at specific cleavage sites^1^. Some cryptic peptides are present also in the brain (neuropeptides, NPs), where they play an important role in the modulation of various processes, such as neuronal signalling, angiogenesis, inflammatory response, immune response, and cell growth^2^. Cryptic peptides may have biological properties and activities that are distinct from their parent proteins^3^^,^^4^.

Opioids are involved in the physiological control of numerous functions of the central nervous system. On the other side, some endogenous NPs, called anti-opioids, participate in the homeostatic system and have the function to contradict the effects of opioids^5^^,^^6^. Particularly, some NPs are generated from the NPFF precursor (pro-NPFF(A)): the NPFF (FLFQPQRF) and the NPAF (AGEGLSSPFWSLAAPQRF), also known as morphine-modulating species. They are members of the FMRF-amide family, which is the largest and most diverse family of NPs known. All peptides are sharing the C-terminal-RF amide sequence^7^ but their mechanism of action is largely unknown. AF and FF peptides can oppose the acute effects of opioids and their elevated concentrations in the brain may be responsible for the development of tolerance and dependence to opioids. Indeed, NPAF and NPFF lower the nociceptive threshold, attenuate morphine analgesia, inhibit stimulated insulin and somatostatin release from the pancreas, exert anorectic properties, inhibit colonic bead expulsion time, regulate body temperature, increase mean arterial blood pressure, control aldosterone release and adrenal blood supply^8^. In addition, they have a large impact also on energy homeostasis by regulating appetite and energy expenditure^9^, demonstrating an important link between RFamide peptides and insulin metabolism^10–12^. We have recently demonstrated that insulin-degrading enzyme (IDE) is able to degrade specific amino acid sequences present in the NP pro-NPFFA, generating some cryptic peptides^1^. It is possible that NPs involved in pain transmission may be partly responsible for the regulation of IDE or IDE indirectly participates in neural communication of pain signals^13^. Intracellular interactions of insulin with IDE may be involved in insulin control of cellular protein degradation and fat oxidation^14–17^. Broad specificity for peptide substrates^18–20^ and its ubiquitous localisation make IDE potentially available for a variety of biomolecular process involved in homeostatic and pathological conditions^21^^,^^22^.

In this scenario, we have applied a recently developed HPLC-MS method^23^ to study the degradation of NPFF and NPAF by IDE, identifying cleavage sites and generated cryptic peptides. Afterwards, we have investigated how NPs and insulin degradation by IDE affect each other and the results hint that the control of IDE activity by allosteric mechanisms could represent a possible link between insulin metabolism and neuronal communication.

## 2. Materials and methods

IDE, His-Tag, rat, recombinant, *Spodoptera frugiperda*, were purchased from Sigma Aldrich. NPAF, NPFF were manually synthesised by the solid-phase method using Fmoc chemistry. Peptides were purified by the high-performance liquid chromatography (HPLC) and tested for their purity using ESI MS. Purity was above 95%. Human insulin, phosphate buffer solution (PBS), trifluoroacetic acid (TFA), acetonitrile (C_2_H_3_N) were purchased from Sigma-Aldrich. The liquid chromatography-electrospray ionisation mass spectrometry (LC-ESI/MS) experiments were carried out by using a LCQ Deca XP instrument (Thermo Electron Corporation, USA). All solutions were incubated in PBS (pH 7.4) at 37 °C. After incubations, sample aliquots at time 0; 10; 30; 45; 60 and 180 min were taken and a 10% solution of TFA was added to the aliquots in order to achieve a final TFA concentration of 1% in all the samples to stop the enzymatic reaction. Samples were kept at 4 °C before HPLC-MS analysis. NPAF or NPFF (20 μM) were incubated with IDE (135 nM), while the concentration of insulin, when used, was 10 μM. In order to assess the effect of NPAF and NPFF fragments on insulin degradation, the peptides were incubated for three hours with IDE and then insulin (10 μM) was added to the reaction mixture. Separation of the generated fragments was carried out by the reversed-phase chromatography using a Jupiter^®^ 5 µm C4 300 Å (LC Column 150 × 4.6 mm) column from Phenomenex at the flow rate of 250 μl/min. MS-MS experiments were used for final identification of the peptide fragments. All methodological details and a semiquantitative method were already described in our previous work, and the absolute intensities of the peaks were normalised to the total ion current^18^^,^^24^^,^^25^. The experiments were repeated three times to ensure reproducibility and estimate the error bars on the HPLC-MS results.

## 3. Results and discussion

**Figure 1.**
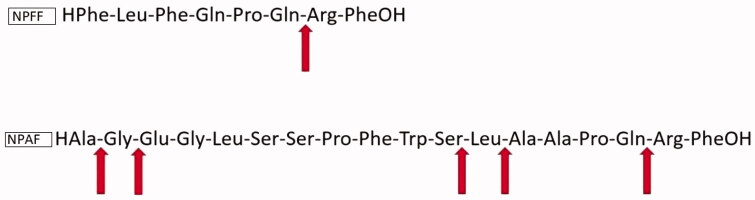
NPFF and NPAF amino acidic sequence. Arrows indicate IDE cleavage sites.

**Figure 2.**
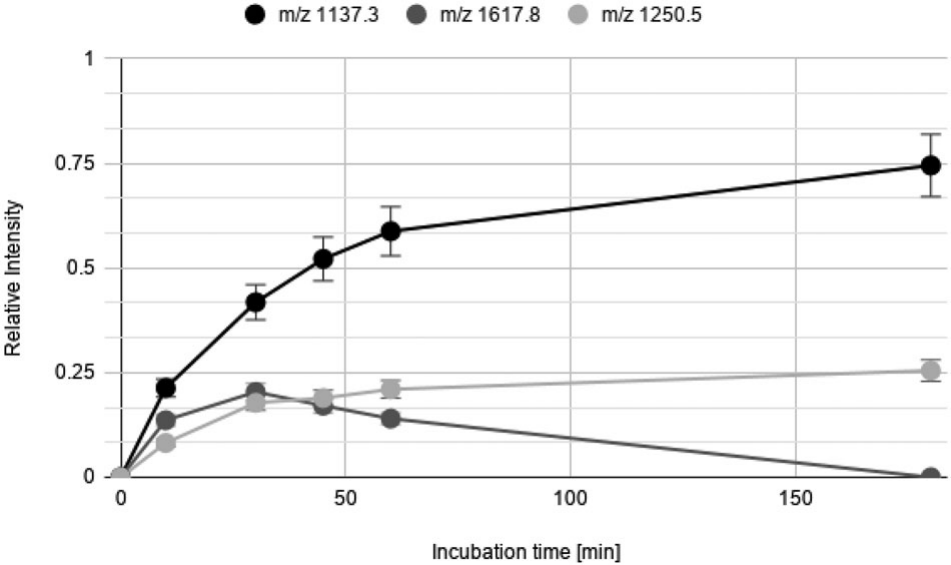
Time courses for the NPAF cryptides formed from the degradation of the NPAF peptide by IDE.

**Figure 3.**
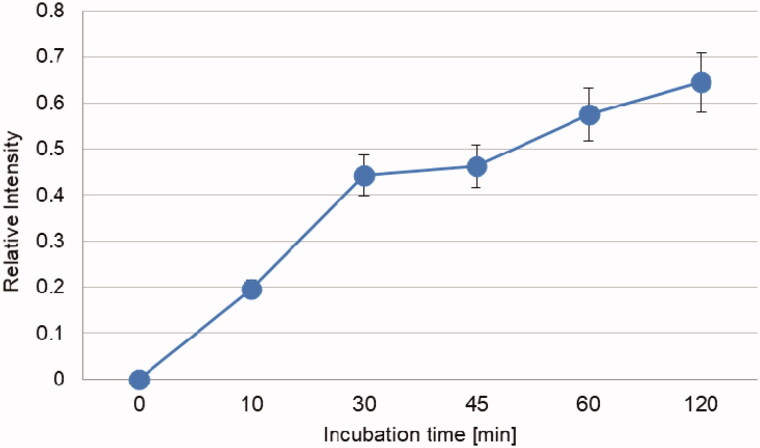
Time courses for the NPFF cryptides at *m/z* 779.3 formed from the degradation of the NPFF peptide by IDE.

In Figure 1, the amino acidic sequences of NPAF and NPFF are reported with arrows indicated the observed IDE cleavage sites, while in Table 1 the assignment of all the peaks detected is also shown. Only the most intense peaks were considered to assess the time courses for the peptides degradation by IDE (Figures 2 and 3). The relative intensity of cryptic peptide at 1617.8 *m/z* for NPAF reaches a maximum after about 30 min of incubation of NPAF with IDE and diminishes its abundance after that time. This trend is easily explained by considering that IDE cleaves peptides in a sequential manner^20^, and therefore longer peptides can be further degraded into shorter fragments by the enzyme soon after their formation. These results clearly demonstrate that IDE is able to cleave both peptides and the observed cleavage sites (Figure 1) are in accordance with the observation that IDE exhibits some preference for basic (Arg) or large hydrophobic (Phe, Leu, Tyr) amino acids at the carboxyl side of the cleavage site^26^.

**Table 1.** Assignment of the peaks observed by ESI-MS for the digestion of NPFF and NPAF solutions by IDE.

Peptide	RT [min]	Calculated (*m/z*)	Experimental (*m/z*)	Sequence
NPFF	**25.6**	**779.4**	**779.3**	**HPhe-Leu-Phe-Gln-Pro-GlnOH**
	26	1082.6	1082.5	HPhe-Leu-Phe-Gln-Pro-Gln-Arg-PheOH
NPAF	**25.9**	**1137.5**	**1137.3**	**HAla-Gly-Glu-Gly-Leu-Ser-Ser-Pro-Phe-Trp-SerOH**
	**26.7**	**1617.8**	**1617.8**	**HAla-Gly-Glu-Gly-Leu-Ser-Ser-Pro-Phe-Trp-Ser-Leu-Ala-Ala-Pro-GlnOH**
	26.8	1663.8	1663.7	HGly-Leu-Ser-Ser-Pro-Phe-Trp-Ser-Leu-Ala-Ala-Pro-Gln-Arg-PheOH
	27.0	1920.9	1921.0	HAla-Gly-Glu-Gly-Leu-Ser-Ser-Pro-Phe-Trp-Ser-Leu-Ala-Ala-Pro-Gln-Arg-PheOH
	27.3	1489.7	1489.8	HGlu-Gly-Leu-Ser-Ser-Pro-Phe-Trp-Ser-Leu-Ala-Ala-Pro-GlnOH
	**27.6**	**1250.6**	**1250.5**	**HAla-Gly-Glu-Gly-Leu-Ser-Ser-Pro-Phe-Trp-Ser-LeuOH**

The most intense peaks, used for assessing the kinetics of peptide degradation, are in bold.

**Figure 4.**
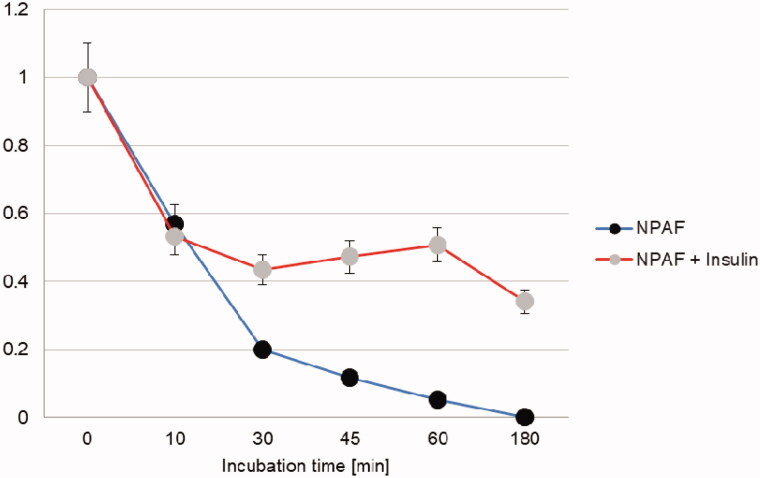
Time course for the NPAF molecular peak decrease observed when the peptide is degraded by IDE in the presence or in the absence of insulin.

**Figure 5.**
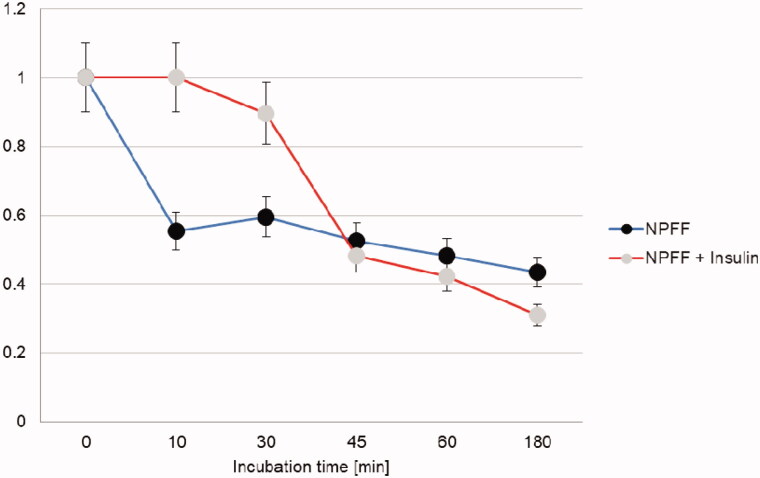
Time course for the NPFF molecular peak decrease observed when the peptide is degraded by IDE in the presence or in the absence of insulin.

Once the degradation of the two peptides by IDE was assessed, the next question that needed a more thorough explanation was how this enzymatic activity affects/is affected by insulin degradation. The latter has been already well characterised in our previous reports^13^^,^^14^^,^^20^ and here we investigated only how the presence of insulin in the incubation mixture affects NPFF and NPAF degradation, and how NPFF and NPAF and their fragments affect insulin degradation by IDE. In order to elucidate the first point, that is how insulin affects NPFF and NPAF degradation, we co-incubated insulin, IDE and NPFF or NPAF together and studied the time courses of the cryptic peptides which are formed. In Figures 4 and 5, the results for NPFF and NPAF are reported, respectively. Strikingly, insulin has a very different effect on the degradation of the two peptides by IDE. Indeed, insulin does not alter NPAF degradation by IDE at short times of incubation, having an inhibitory effect on the peptide degradation only after several minutes of co-incubation. Considering that insulin degradation and the formation of insulin fragments by IDE are very quick at these experimental conditions^20^, the observed results can be explained by assuming that the insulin fragments, rather than the full-length hormone, exert an inhibitory effect on NPAF degradation by IDE. On the contrary, insulin inhibitory effect observed in the case of NPFF at very short incubation times indicates that, in this case, it is the full-length insulin, which is slowing down the peptide degradation, whereas the insulin fragments have little effect on IDE degrading activity towards NPFF. For a peptide to be degraded by IDE, the former must bind to the enzyme, access its catalytic site containing a zinc ion, form an intermediate complex, and finally fragments will be released. If there are more than one IDE substrates in solution (as in our case with the coexistence of insulin and the NPs), it is conceivable that IDE will degrade first and more quickly the peptides which have a higher binding affinity for the enzyme. We have already reported that longer NPs have higher affinity for IDE than shorter ones^1^ and NPFF is much shorter than NPAF (Figure 1). From this perspective, our different results on the inhibitory role of insulin on cleavage of the two peptides can be easily explained by assuming that, also in this case, NPFF has lower affinity for the enzyme. Therefore, in a solution containing insulin and NPFF, IDE will first bind and degrade insulin. Longer incubation times will be needed for IDE to degrade appreciably the NPFF present in solution with the insulin. On the contrary, the larger dimensions of NPAF make this peptide a more suitable candidate to bind IDE competitively also in the presence of insulin, therefore, when NPAF and insulin coexist in solution, IDE cleaves both very efficiently. Of course, as time goes by and the solution becomes rich of insulin and peptide fragments, a competition for the occupation of the enzyme catalytic site occurs and a decrease of NPAF degradation at longer incubation time is observed (Figure 4).

**Figure 6.**
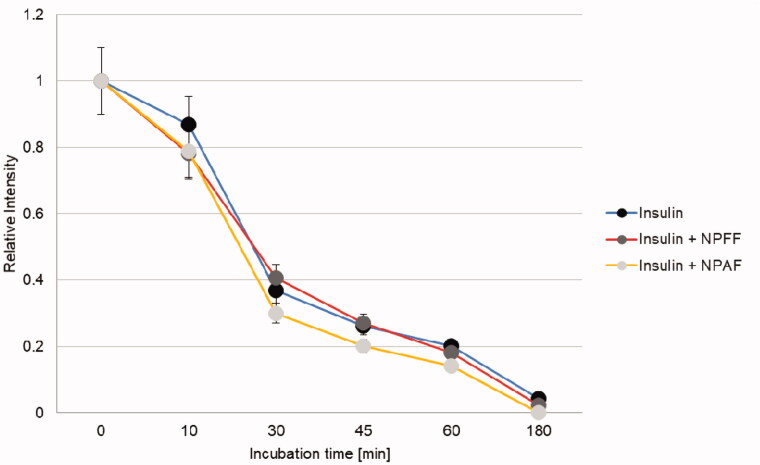
Time course for the insulin molecular peak decrease observed when the hormone is degraded by IDE in the presence or in the absence of NPAF/NPFF.

A further insight into the reciprocal effect that insulin and the NPs have on IDE proteolytic activity can be obtained by following insulin degradation by IDE in the presence of the NPs. In Figure 6, the relative intensities of the insulin molecular peak as a function of time is plotted for solutions containing IDE and insulin or a mixture of IDE, insulin and the NPs. In this case, although the curves are all very similar to each other, a small but reproducible (experiments were performed three times) activating effect for NPAF on insulin degradation by IDE is observed (faster degradation of the intact insulin). It is well known that small molecules and peptides can be activators of IDE through their binding to allosteric sites^27^^,^^28^ and our results indicate that NPAF but not NPFF can be an IDE activator as well as a substrate. In addition, we performed experiments co-incubating IDE and NPs for 3 h prior the addition of insulin, in order to see if the small activation effect observed in the case of NPAF can be attributed to the full-length peptide or to an IDE generated cryptide. We observed no activation effect in this case as all the curves recorded (incubation for 3 h of IDE with and without NPAF/NPFF and then addition of insulin) are superimposable (data not shown). This means that the small activation effect observed in the case of NPAF is due to the full-length peptide and not to any of its IDE produced cryptides.

**Figure 7.**
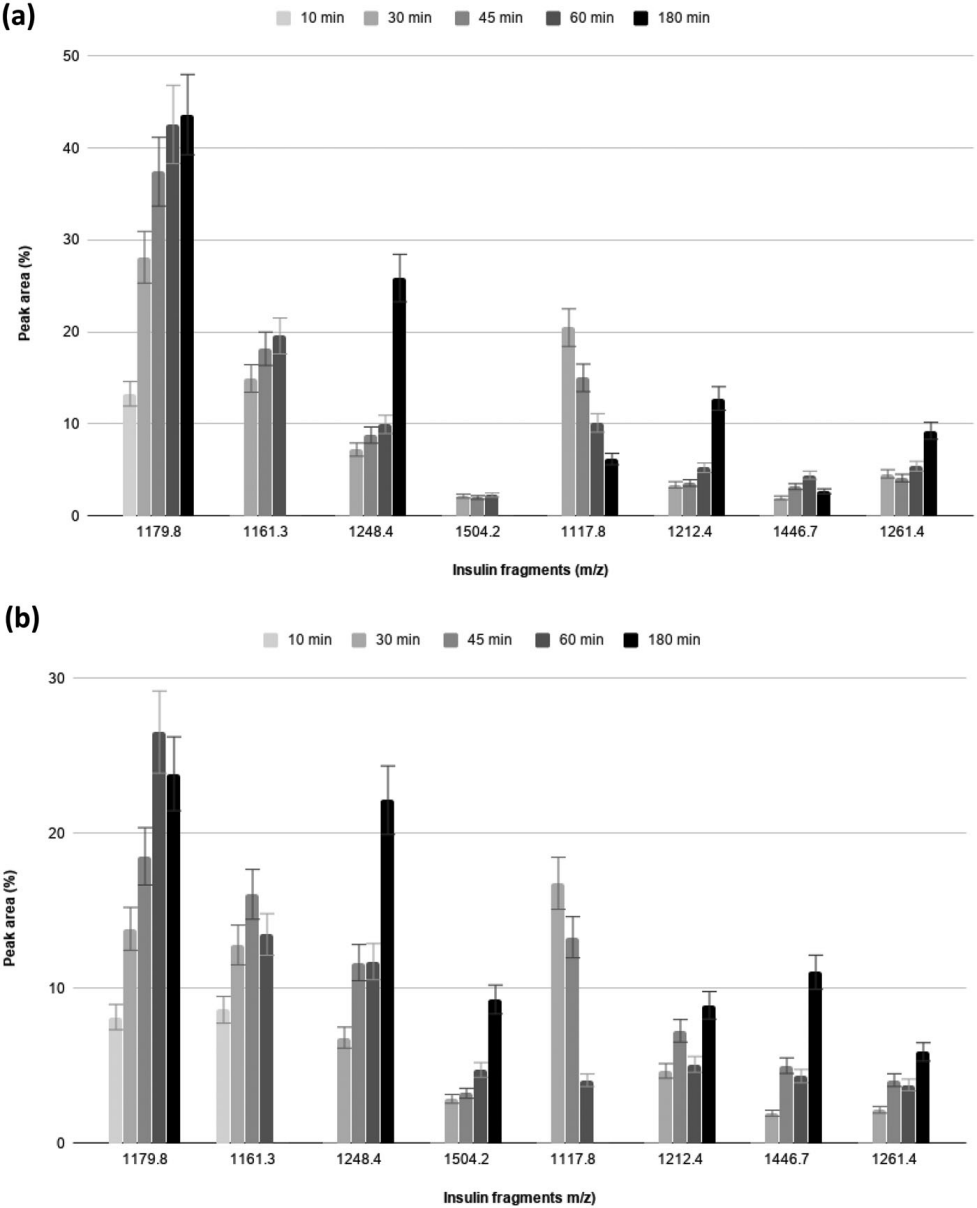
Hydrolytic patterns of time-courses insulin degradation by IDE in the absence (a) and in the presence (b) of NPAF.

In order to see if the NPAF small activatory effect on insulin degradation by IDE is site specific, and therefore leads to an increase in the amount of specific insulin cryptides rather than to an overall aspecific increase of insulin degradation, we have monitored the relative intensities of all the insulin fragments versus time of incubation^23^. This investigation is important because specific insulin fragments can have different biological activities *in vivo*^14^. In Figure 7, the time courses of the most intense IDE produced insulin fragments are reported, for solutions containing IDE and insulin with and without NPAF. It is shown that for some insulin fragments [A(14–21) B(14–24), A(1–14) B(14–30) and A(1–14) B(14–25), see Table 2] the detected time courses are evidently altered in the presence of NPAF, while others remain unaffected. Particularly, the analysis of the fragments whose abundance is increased by the presence of NPAF, indicates that the latter increases IDE activity towards insulin in a site-specific manner, favouring the action of the enzyme mainly at the B13–14 insulin cleavage site. This result hints that the interaction of IDE with NPAF somehow must modify the binding of insulin with the enzyme, so that the latter increases its catalytic activity towards a specific portion of the insulin amino acidic sequence.

**Table 2.** Assignment of the peaks observed by ESI-MS for the digestion of insulin solutions by IDE.

Detected insulin fragments	Calculated monoisotopic peaks (*m/z*)	Experimental monoisotopic peaks (*m/z*)
A(1–13) B(1–9)	1179.8	1179.3
A(1–14) B(1–9)	1261.4	1261.4
**A(14–21) B(14–24)**	**1504.2**	**1504.5**
A(1–13) B(1–10)	1248.4	1248.8
A(1–13) B(17–24)	1117.8	1117.5
**A(1–14) B(14–30)**	**1161.3**	**1161.8**
A(1–13) B(17–25)	1212.4	1212.8
**A(1–14) B(14–25)**	**1446.7**	**1447.0**

The fragments generated by IDE whose relative abundance increases in the presence of NPAF are indicated in bold. Discussion is in the text.

## 4. Conclusions and future perspectives

We have investigated the degrading activity of IDE for some NPs, which have been demonstrated to lower the nociceptive threshold, functioning as anti-opioids, as well as inhibiting insulin release from the pancreas. The reported results clearly indicate that IDE is capable of degrading both NPAF and NPFF at specific cleavage sites. Moreover, our results indicate that the degradation of the two NPs is differently affected by the presence of insulin. Indeed, while NPFF degradation is slowed down by the presence of the full-length insulin, the degradation of the longer NPAF is unaffected by the presence of the intact hormone, whereas generation of shorter insulin fragments produced by the action of IDE is responsible for the inhibition of the NPs degradation at longer incubation times. We explain this difference in the inhibitory role of insulin towards the two NPs by considering their different length, and thus structure, which very likely is the cause of a different affinity for the enzyme. An SPR investigation to confirm such hypothesis will be the subject of future work.

Finally, we have also demonstrated that NPAF, but not NPFF, plays an activatory role on the degradation of insulin by IDE in a site-specific manner, favouring the action of the enzyme mainly at the B13–14 insulin cleavage site. Once again, this result demonstrates the pivotal role of IDE in the interplay between NPs and insulin metabolism, giving a molecular insight into the link between type 2 or type 1 diabetes and pain transmission and pain threshold^13^^,^^29^.
